# Exosomal circRNA Transcriptome Analysis of Porcine Intestinal Epithelial Cells Under *E. coli* F18 Infection

**DOI:** 10.3390/ani16172684

**Published:** 2026-08-27

**Authors:** Yifu Wang, Yihan Chen, Ming-An Sun, Zhengchang Wu

**Affiliations:** 1 College of Veterinary Medicine, Yangzhou University, Yangzhou 225009, China; 2 College of Animal Science and Technology, Yangzhou University, Yangzhou 225009, China

**Keywords:** pig, exosomes, circRNAs, *E. coli* F18 susceptibility

## Abstract

Enterotoxigenic *Escherichia coli* F18 (*E. coli* F18) ranks as the leading bacterial cause of diarrhea in piglets. Exosomes and other extracellular vesicles ferry diverse RNA species, among them mRNAs and non-coding RNAs, and act as central intermediaries in intercellular communication. Their contribution to the biology of *E. coli* F18-infected IPEC-J2 cells, however, has received no attention. In this study, we characterized the morphology, particle dimensions, and output of exosomes secreted by control and *E. coli* F18-infected cells, then profiled their circRNA cargo by RNA-seq. Nanoparticle tracking analysis (NTA), transmission electron microscopy (TEM), and Western blotting verified that the vesicle-like particles recovered from IPEC-J2 cultures were bona fide exosomes. We then catalogued the circRNAs within IPEC-J2-derived exosomes during *E. coli* F18 infection. Comparing the two groups yielded 280 differentially expressed circRNAs (DECs), of which circCHUK displayed the largest shift. circCHUK abundance rose sharply in *E. coli* F18-treated IPEC-J2 cells. Knocking down or overexpressing circCHUK markedly altered the adherence of *E. coli* F18 to IPEC-J2 cells. ceRNA network construction pinpointed the exo-circCHUK/ssc-miR-339-3p/FUT2 as candidate axis. Collectively, we speculated that exo-circCHUK is a new biomarker that regulates *E. coli* F18 susceptibility, offering a conceptual framework for tackling bacterial diarrhea in piglets.

## Simple Summary

Diarrhea triggered by enterotoxigenic *E. coli* F18 after weaning inflicts considerable economic damage on the pig industry. As extracellular vesicles, exosomes participate in cell-to-cell signaling and show promise as biomarkers for various autoimmune conditions. However, no prior work has examined the biological activities or potential clinical significance of circRNAs in IPEC-J2 cells infected with *E. coli* F18. Our results indicated the exo-circCHUK/miR-339-3p/FUT2 as a probable candidate axis of how susceptible IPEC-J2 cells are to *E. coli* F18 infection. To our knowledge, this is the first report to define the role and molecular basis of exosomal circRNAs in governing *E. coli* F18 susceptibility, laying groundwork for delineating the pathogenic mechanisms of *E. coli* F18 and for uncovering novel antibacterial targets.

## 1. Introduction

Pig producers incur heavy financial losses from post-weaning diarrhea (PWD), a disease shaped by numerous interacting variables. Bacterial and viral infections, nutritional imbalances, inadequate hygiene, management practices, and environmental factors such as humidity and temperature fluctuations all contribute. Among the infectious agents responsible for gastrointestinal illness in intensive swine operations, enterotoxigenic *Escherichia coli* F18 (*E. coli* F18) is one of the most prevalent. Although these infections are seldom lethal compared with viral diarrhea, they can still depress growth performance, compromise feed conversion, and reduce overall profitability [1]. The genetic basis underlying piglet susceptibility to *E. coli* F18 infection, however, remains poorly defined.

Circulating extracellular vesicles hold considerable promise as biomarkers for autoimmune disease diagnosis, prognosis, and therapeutic monitoring [2,3,4]. Exosomes in particular encapsulate a varied payload: both messenger RNA and non-coding RNA classes (miRNA, lncRNA, and circRNA), together with proteins that mediate intercellular signaling and communication upon delivery to recipient cells [5]. In one notable example, exosomal miR-149-3p from enterotoxigenic *Bacteroides fragilis* (ETBF)-treated cells was shown to drive T-helper type 17 cell differentiation [6]. These observations raise the possibility that miRNAs packaged in exosomes may function as diagnostic indicators of bacterial infection. To date, however, no study has systematically profiled the circRNA and mRNA complements of exosomes from *E. coli* F18-infected cell supernatants or examined how these RNA classes interact.

Found across a broad range of organisms, from humans and other animals to plants, circular RNAs (circRNAs) are non-coding transcripts distinguished by a covalently closed loop that lacks both a 5′ cap and a 3′ tail, features tied to their varied regulatory activities inside cells [7]. In pigs (*Sus scrofa*), a species valued as both a biomedical model and a major agricultural resource, circRNA biology is just beginning to be explored. One established mechanism is miRNA sponging: circRNAs bind microRNAs (miRNAs) and sequester them, blocking the miRNAs from repressing their target mRNAs and thereby lifting gene expression indirectly [8]. Within pigs, individual circRNAs have been linked to the control of genes underlying muscle development, fat deposition, and other production-relevant physiological traits [9,10,11]. Immune defense and disease resistance also fall within the functional scope of circRNAs in this species [12,13,14]. Porcine circRNAs shift in expression when animals encounter pathogens such as porcine reproductive and respiratory syndrome virus (PRRSV), pointing to a role in calibrating the host immune response [15]. However, the functions of circRNAs in susceptibility to *E. coli* F18 in intestinal porcine epithelial (IPEC-J2) cells are largely unknown. In this study, we aim to isolate and characterize the exosomes from *E. coli* F18-infected and uninfected IPEC-J2 cell supernatants, and to examine the cellular exosome morphology, particle size, and secretory concentration. A comparative circRNA transcriptome analysis of exosomes from infected cells was then performed to identify circRNAs governing *E. coli* F18 susceptibility. Bioinformatic tools were used to assemble a competing endogenous RNA (ceRNA) network around candidate circRNAs, clarifying their possible mechanistic contributions to infection. Taken together, these findings advance understanding of how circRNA expression relates to *E. coli* F18 susceptibility and provide a basis for molecular strategies to strengthen weaned piglet resistance against this economically damaging pathogen.

## 2. Materials and Methods

### 2.1. Ethics Statement

Yangzhou University granted ethical approval for all experimental protocols (Pig: SYXK (Su) 2012-0029), and every procedure complied with the ethical standards of the People’s Republic of China.

### 2.2. Animals

Meishan piglets previously characterized as *E. coli* F18-resistant or -sensitive were used for in vivo validation of candidate circRNA expression. Meishan piglets classified as *E. coli* F18-sensitive or -resistant were identified by challenged with *E. coli* F18 strain and then performing bacterial counting, histopathological examination, and in vitro adherence assays [16]. Each piglet was housed individually in separate pens. They were fed ad libitum with a commercial-type compound feed for weaned piglets containing 21.7% crude protein, without antimicrobial additives and organic acids. Beginning at day 3 post-weaning, experimental piglets were challenged with a daily dose of 4.6 × 10^8^ CFU of *E. coli* F18 strain once a day for up to 10 days, or until they showed diarrhea. Among these, we selected three resistant and three sensitive female piglets at 35 days of age, from the same litter, with almost same birth weight and weaning weight. Piglets were euthanized with an intravenous injection of pentobarbital sodium. Jejunal and duodenal tissues were preserved in liquid nitrogen for downstream analysis.

### 2.3. Cellular Culture and Treatment

IPEC-J2 cells, originally obtained from the University of Pennsylvania (Philadelphia, PA, USA), were maintained in DMEM supplemented with 10% FBS. Cells were seeded into 12-well plates at a density of 5.0 × 10^5^ cells/well and incubated under standard conditions (37 °C, 5% CO_2_). Once cultures reached roughly 80% confluence (after 24 h), stimulation was performed using either *E. coli* F18ab [107/86 (O139:K12:H1)] or *E. coli* F18ac [2134P (O157:H19)] at 1.0 × 10^9^ CFU/mL, 0.1 μg/mL LPS (Merck KGaA, Darmstadt, Germany), or plain medium serving as the vehicle control. Each treatment group included three biological replicates. RNA was harvested at 0, 2, 4, and 6 h after lipopolysaccharide (LPS) exposure, whereas *E. coli*-stimulated cells were collected at 4 h post-treatment.

### 2.4. Indirect Immunofluorescence (IFA)

PBS washes were performed on IPEC-J2 cells (both *E. coli* F18-treated and control groups) before fixation with 4% paraformaldehyde and permeabilization using Triton X-100 (Merck KGaA, Darmstadt, Germany). Goat blocking serum was applied for 1 h. Cells were then probed with an *E. coli* antibody (Abcam, Cambridge, UK, 1:500) and subsequently with IgG-HRP secondary antibody (Abcam, Cambridge, UK, 1:3000). Nuclear counterstaining was done with DAPI (Abcam, Cambridge, UK, 1:1000), after which the cells underwent three PBS washes, were mounted using fade-resistant fluorescent mounting medium, and imaged under a confocal microscope.

### 2.5. Isolation and Identification of Exosomes

Exosome isolation followed a published protocol [17]. Culture medium was harvested at 24 h, and exosomes were purified from supernatants of both *E. coli* F18-infected (*E. coli* F18-Exos) and normal (Control-Exos) IPEC-J2 cells through sequential centrifugation steps designed to clear cells and debris: 2000× *g* for 30 min, 10,000× *g* for 45 min, and 100,000× *g* for 70 min. Filtration through a 0.22-µm membrane (Millipore, Billerica, MA, USA) followed. The filtered supernatant underwent ultracentrifugation at 150,000× *g* for 90 min at 4 °C (Optima™ XPN, Beckman Coulter, Brea, CA, USA) to pellet the exosomes. Each pellet was resuspended in 1 mL sterile PBS and passed through a 0.22-µm syringe-driven filter unit (Millipore). Aliquots of 200 µL were stored at −80 °C pending downstream use. Western blotting against the exosomal markers TSG101 (14497-1-AP, Proteintech, Chicago, IL, USA, 46 kDa), CD9 (20597-1-AP, Proteintech, 26 kDa), CD63 (DF2305, Affinity, Nottingham, UK, 26 kDa), and CD81 (ab109201, Abcam, 26 kDa), along with GAPDH (60004-1-IG, Proteintech, 46 kDa), was used to verify exosome identity. NTA (Particle Metrix, Meerbusch, Germany) and TEM (Hitachi, Tokyo, Japan) provided additional morphological and size characterization.

### 2.6. Total DNA and RNA Extraction, cDNA Synthesis

Total DNA was extracted from IPEC-J2 cells and from jejunal and duodenal tissue samples using a commercial DNA extraction kit (Beijing Tiangen Biochemical Co., Ltd., Beijing, China). DNA concentration and purity were checked on a NanoDrop 1000 (Thermo Fisher Scientific, Waltham, MA, USA) instrument and by 2% agarose gel electrophoresis. For RNA isolation, TRIzol reagent (Invitrogen, Carlsbad, CA, USA) was applied to the same sample types (jejunal tissue, duodenal tissue, and IPEC-J2 cells), and RNA quality was again evaluated on a NanoDrop 1000 (Thermo Fisher Scientific, Waltham, MA, USA). cDNA synthesis was carried out using a reverse transcription kit (Vazyme Co., Ltd., Beijing, China) in 10 µL reactions containing 500 ng RNA, 2 µL of 5× RT Master Mix II, and ddH_2_O. Reverse transcription was performed at 25 °C for 10 min, 50 °C for 30 min, and 85 °C for 5 min, and the resulting cDNA was held at 4 °C.

### 2.7. qPCR Analyses

qPCR reactions relied on AceQ qPCR SYBR Green Master Mix (Vazyme Biotech Co., Ltd., Nanjing, China) together with the primers listed in Table S1 (Sangon Biotech Co., Ltd., Shanghai, China). Each 20 µL reaction was assembled with 2 µL cDNA, 0.4 µL each of F/R primers (10 µM), 10 µL of 2× AceQ qPCR SYBR Green, 0.4 µL of 50× ROX Reference Dye I, and ddH_2_O. Cycling began with an initial 95 °C hold for 5 min, followed by 40 cycles of 95 °C (10 s) and 60 °C (34 s). A melt curve program of 95 °C for 10 s, 60 °C for 60 s, and 95 °C for 15 s concluded the run.

### 2.8. Western Blotting

Protein samples were resolved on 10% SDS-PAGE gels and electroblotted onto polyvinylidene difluoride membranes. Following blocking, membranes received overnight primary antibody incubation at 4 °C. Detection employed a horseradish peroxidase-conjugated secondary antibody (Sigma-Aldrich, St. Louis, MI, USA) together with enhanced chemiluminescence reagents.

### 2.9. RNA Silencing and Overexpression

Gene Pharma Co., Ltd. (Shanghai, China) synthesized three siRNA sequences targeting circCHUK (si-circCHUK-1/2/3), the matching negative controls (si-NC; primers in Table S2), an OE-circCHUK overexpression construct, and its negative control vector (pGLV5-NC). Transfection of IPEC-J2 cells was carried out with Lipofectamine 2000 (Thermo Fisher Scientific, Waltham, MA, USA), using three biological replicates for each group. Knockdown and overexpression efficiencies were confirmed by qPCR.

### 2.10. CircRNA Transcriptome Sequencing

Total RNA was extracted from exosomes purified from control (NC, *n* = 3) and *E. coli* F18-infected (*E. coli*, *n* = 3) IPEC-J2 supernatants and reverse-transcribed into double-stranded cDNA. Oebiotech Corporation (Shanghai, China) carried out sequencing on an Illumina HiSeq 2500 platform (Illumina, San Diego, CA, USA). CircRNA transcriptome profiling followed the manufacturer’s protocol, and downstream data analysis proceeded as described in a prior report [18].

### 2.11. Prediction of circRNA–miRNA–mRNA Networks

Putative miRNA targets of the differentially expressed circRNAs were identified by combining two databases: circNetVis (https://www.meb.ki.se/shiny/truvu/CircNetVis/, accessed on 20 January 2026) [19] and miRDB (http://mirdb.org, accessed on 20 January 2026) [20]. miRNA-mRNA pairings were then forecast using OECloud tools (https://cloud.oebiotech.com, accessed on 25 January 2026) together with TargetScan (targetscan.org) [21]. Target-prediction parameters were set as canonical 7–8 mer seed, MFE ≤ −20 kcal/mol and miRanda score ≥ 140. Cytoscape 3.9.1 software was used to map miRNA-mRNA and miRNA-lncRNA interactions into a ceRNA regulatory network [22].

### 2.12. E. coli Adherence Assay

Fimbrial adhesion of *E. coli* F18ab/ac to IPEC-J2 monolayers in vitro was assessed using an established protocol [23]. *E. coli* F18 strains were grown in LB medium on a rocking platform (200 r/min, 12 h). Approximately 5 × 10^5^ IPEC-J2 cells per well in 12-well plates (Corning, Corning, NY, USA) were then challenged with 1 × 10^9^ CFU of *E. coli* for 3 h at 37 °C. Cultures were centrifuged at 4000 rpm for 5 min, after which the two *E. coli* supernatant types were sterile-filtered (0.22 μm pore size). Adhesive capacity was evaluated through colony counting and indirect immunofluorescence, following the approach of Dai et al. (2019) [24]. Pili, also known as fimbriae, are proteinaceous, filamentous polymeric organelles expressed on the surface of bacteria. Pili are composed of single or multiple types of protein subunits, called PILIN, which are typically arranged in a helical fashion. A relative quantification assay for the *E. coli* fimbrial protein PILIN [25] provided an additional measure of adhesion.

### 2.13. Statistical Analyses

qPCR quantification used the 2^−ΔΔCt^ method with GAPDH as the internal reference gene. Group comparisons of data (presented as means ± SD) were carried out in SPSS 25.0 software (SPSS, Inc., Chicago, IL, USA). The *p*-values were calculated using Student’s *t*-tests or one-way analysis of variance. *p* < 0.05 was considered to be statistically significant. All treatments were performed with three biological replicates.

## 3. Results

### 3.1. Identification and Characterization of Exosomes from Control and E. coli F18-Infected IPEC-J2 Cell Supernatants

**Figure 1:**
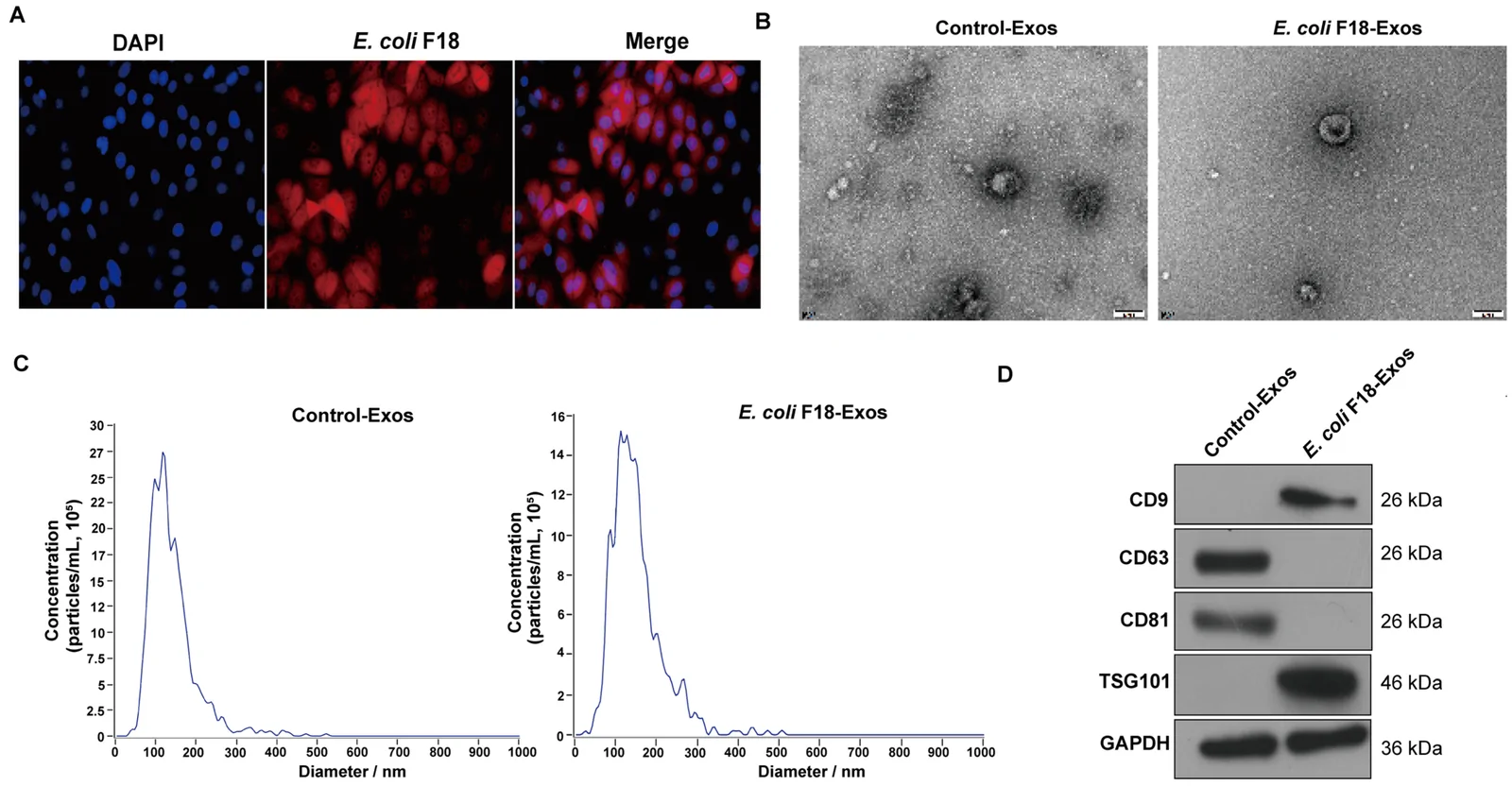
Verification and phenotypic profiling of exosomes harvested from culture supernatants of *E. coli* F18-infected IPEC-J2 cells. (**A**) Establishment of the *E. coli* F18-infected IPEC-J2 cell model, validated by indirect immunofluorescence. (**B**) Representative transmission electron microscopy images showing the morphology of supernatant-derived exosomes. Scale bar: 100 nm. (**C**) Particle size distribution of the isolated exosomes, measured by nanoparticle tracking analysis. (**D**) Western blot confirmation of canonical exosomal markers CD63, CD9, CD81, and TSG101.

Indirect immunofluorescence (Figure 1A) was used to confirm the establishment of an *E. coli* F18-infected IPEC-J2 cell model, from whose supernatants exosomes were then recovered by ultracentrifugation. Vesicle identity was verified through TEM, NTA, and Western blotting. Under TEM, the isolated particles displayed the round, bilayer-membrane morphology and roughly 100 nm diameter typical of exosomes (Figure 1B). NTA placed the majority of the isolated IPEC-J2-derived vesicles in the 50 to 250 nm size range (Figure 1C), consistent with established exosome dimensions. Western blotting confirmed the presence of the exosomal markers CD9, CD63, CD81, and TSG101. CD9 and TSG101 were detected in exosomes derived from *E. coli* F18-infected IPEC-J2 cells, while CD9 and CD81 were present in exosomes from uninfected IPEC-J2 cells (Figure 1D). Taken together, these findings confirm the exosomal identity of the isolated vesicles.

### 3.2. Identification and Characterization of circRNA Expression in Exosomes

To explore the presence of circRNAs in exosomes from control and *E. coli* F18-infected IPEC-J2 cell supernatants, we prepared and sequenced ribo-depleted total RNAseq libraries (Table S3). On average, approximately 96.3 and 94.8 million reads were obtained from three independent biological replicates of *E. coli* F18-infected group (*E. coli*) and control group (NC). Across all six datasets, the valid read ratio exceeded 92% and Q30 quality scores surpassed 90%, confirming reliable sequencing depth and accuracy.

**Figure 2:**
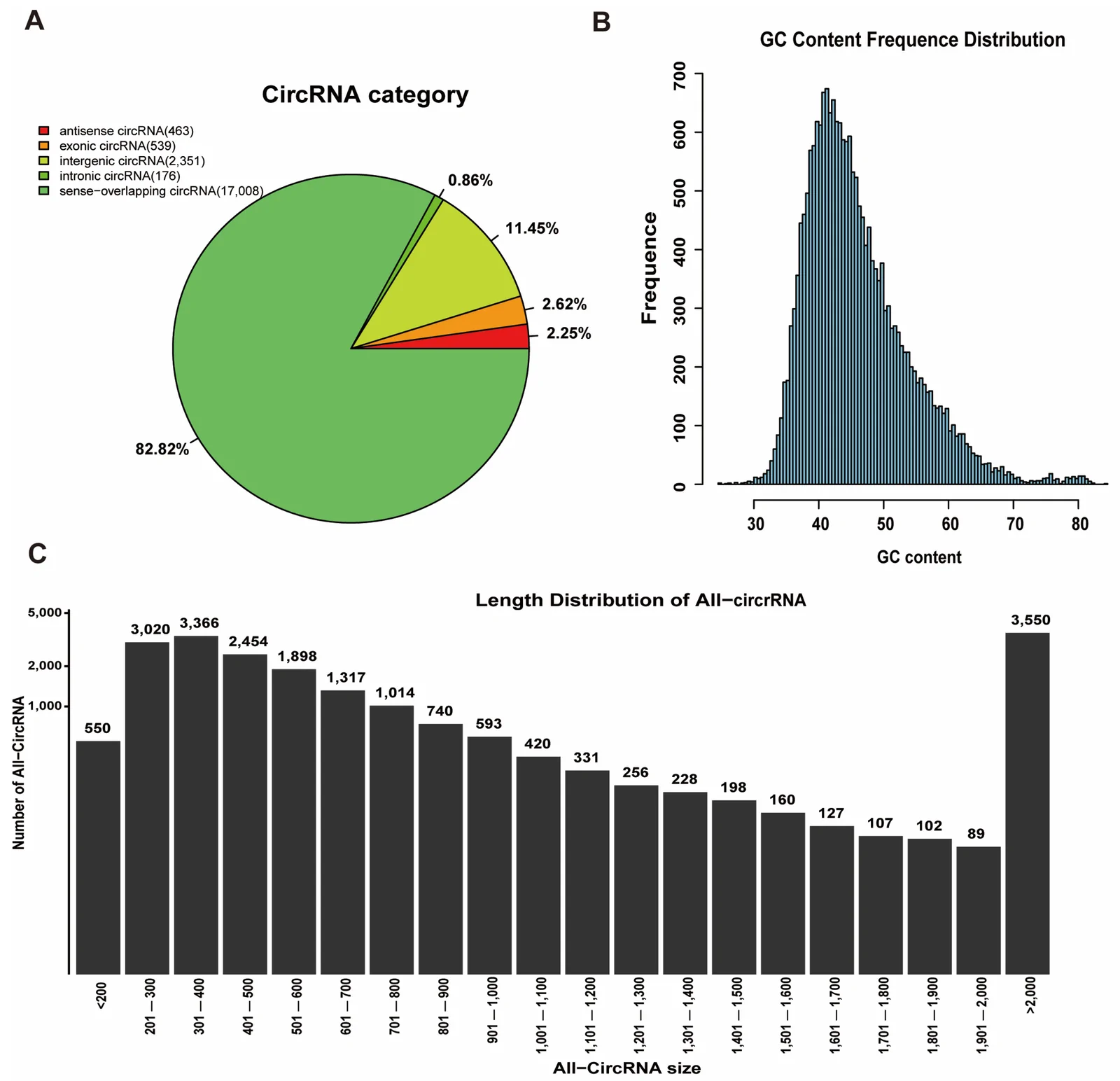
Global profile of circRNAs sequenced from exosomes of the *E. coli* and NC groups. (**A**) Categorization of circRNAs mapped to the *Sus scrofa* genome. (**B**) Distribution of GC content across detected circRNAs. (**C**) Length distribution of the identified circRNAs.

Sequence analysis was used to profile circRNAs in exosomes from both the *E. coli* and NC groups. A catalog of 20,537 candidate circRNAs was assembled, each supported by at least one unique back-spliced read. These molecules fell into five structural classes: antisense, exonic, intergenic, intronic, and sense-overlapping circRNAs. Sense-overlapping circRNAs dominated the set (17,008 transcripts, 82.82%), with intergenic circRNAs ranking second (2351, 11.45%). Exonic (539, 2.62%) and antisense (463, 2.25%) circRNAs accounted for only a minor fraction (Figure 2A). GC content for the majority of these circRNAs ranged from 35 to 60% (Figure 2B). Genomic mapping revealed broad distribution across all chromosomes (Figure 2B), and nearly all transcripts (19,987, 97.14%) exceeded 200 nt (Figure 2C).

### 3.3. Identification of Differentially Expressed circRNAs in Exosomes of IPEC-J2 with E. coli F18 Infection

**Figure 3:**
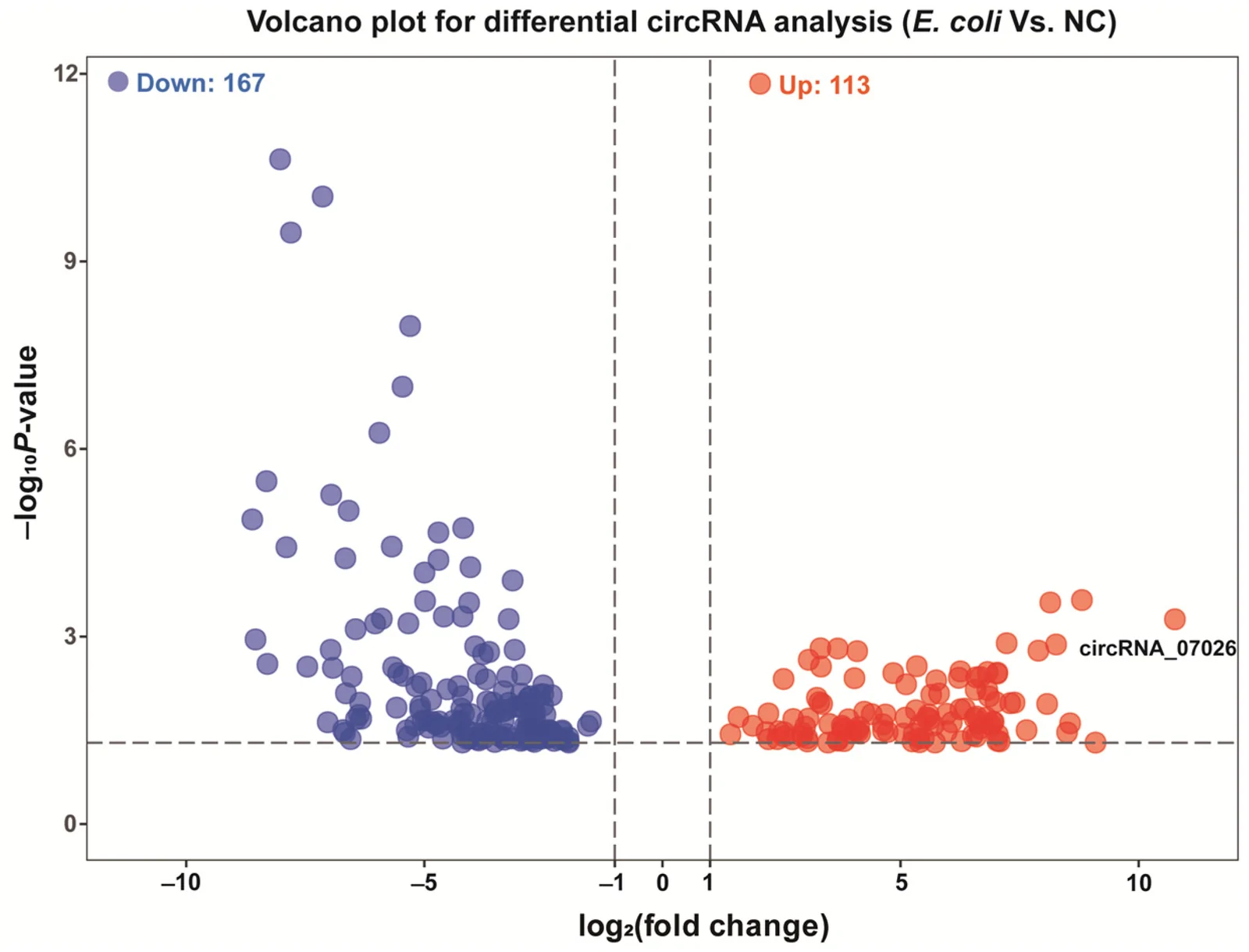
Volcano plot showing DECs in exosomes from the *E. coli* and NC groups. Red dots correspond to DECs upregulated in the *E. coli* group, while blue dots correspond to downregulated DECs. Significance thresholds were set at |log_2_FC| > 1 and *p* < 0.05.

To identify the expression profile of circRNAs in exosomes of IPEC-J2 with *E. coli* F18 infection, we performed a comparative circRNA transcriptome analysis of supernatant exosomes from 3 normal IPEC-J2 and 3 *E. coli* F18-infected IPEC-J2. Applying thresholds of FC ≥ 2 and adjusted *p* ≤ 0.05 identified 280 differentially expressed circRNAs (DECs). Of these, 113 were upregulated and 167 were downregulated in the *E. coli* group relative to controls (Figure 3, Table S4).

### 3.4. GO and KEGG Pathway Enrichment Analyses of Differential circRNA Parental Genes

**Figure 4:**
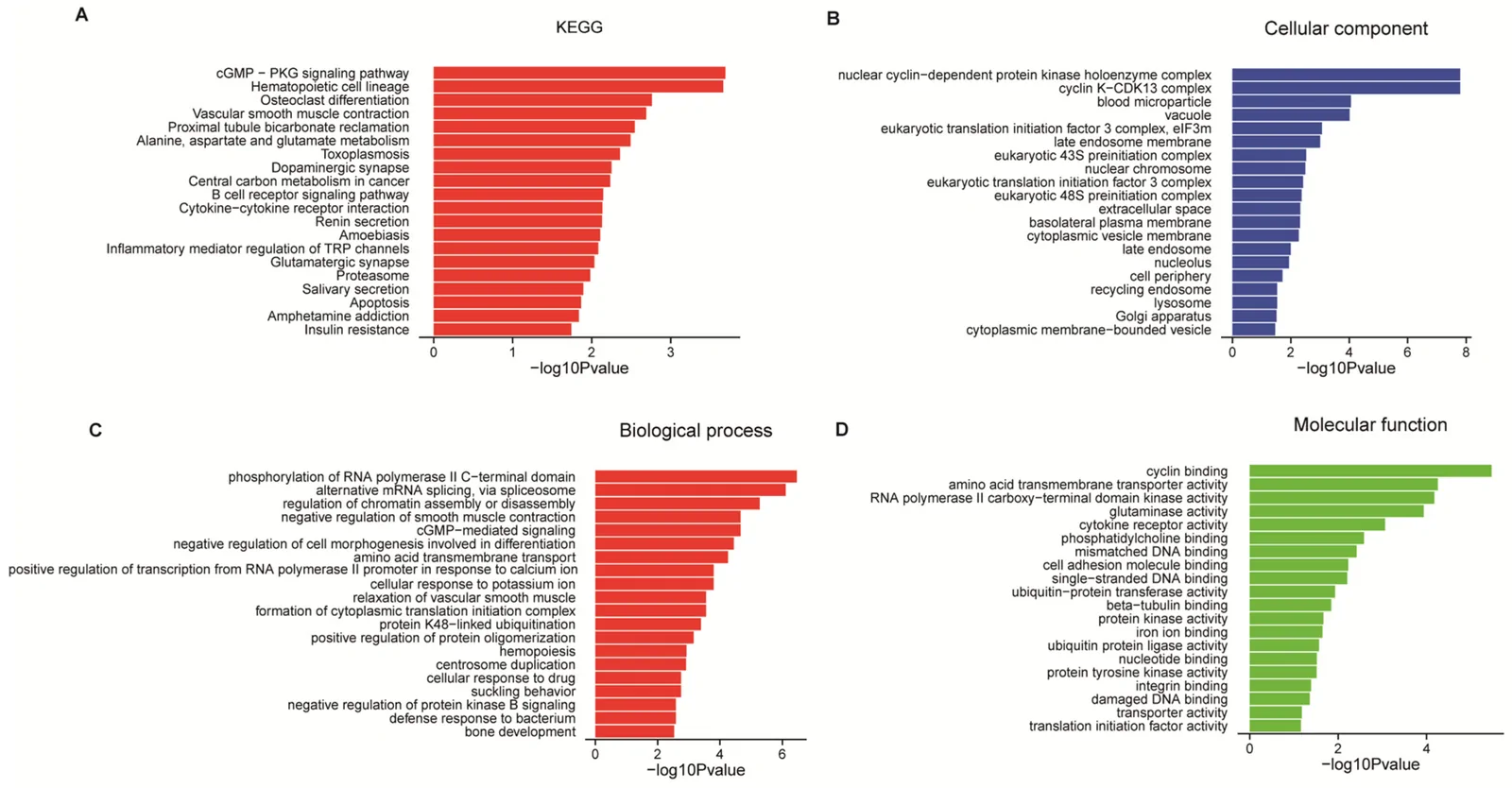
GO term and KEGG pathway enrichment analyses of differential circRNA parental genes. (**A**) Enrichment analysis of the KEGG pathway of circRNA parental genes. (**B**–**D**) GO functional annotation of differential circRNA parental genes.

Because circRNA function is tightly linked to that of their parental genes [26], GO annotation and KEGG pathway enrichment were applied to the parental genes of the DECs to infer potential mechanisms. KEGG analysis pointed to enrichment in the cGMP-PKG signaling pathway (ssc04022) and Hematopoietic cell lineage (ssc04640), along with immune-related pathways including Cytokine–cytokine receptor interaction (ssc04060) and the B cell receptor signaling pathway (ssc04662) (Figure 4A, Table S5). GO terms indicated association with the cyclin K-CDK13 complex (GO:0002945) and the nuclear cyclin-dependent protein kinase holoenzyme complex (GO:0019908) (Figure 4B), as well as phosphorylation of the RNA polymerase II C-terminal domain (GO:0070816), alternative mRNA splicing via the spliceosome (GO:0000380) (Figure 4C), and cyclin binding (GO:0030332) (Figure 4D).

### 3.5. Characterization of the Association Between Key CircRNA and E. coli F18 Infection

**Figure 5:**
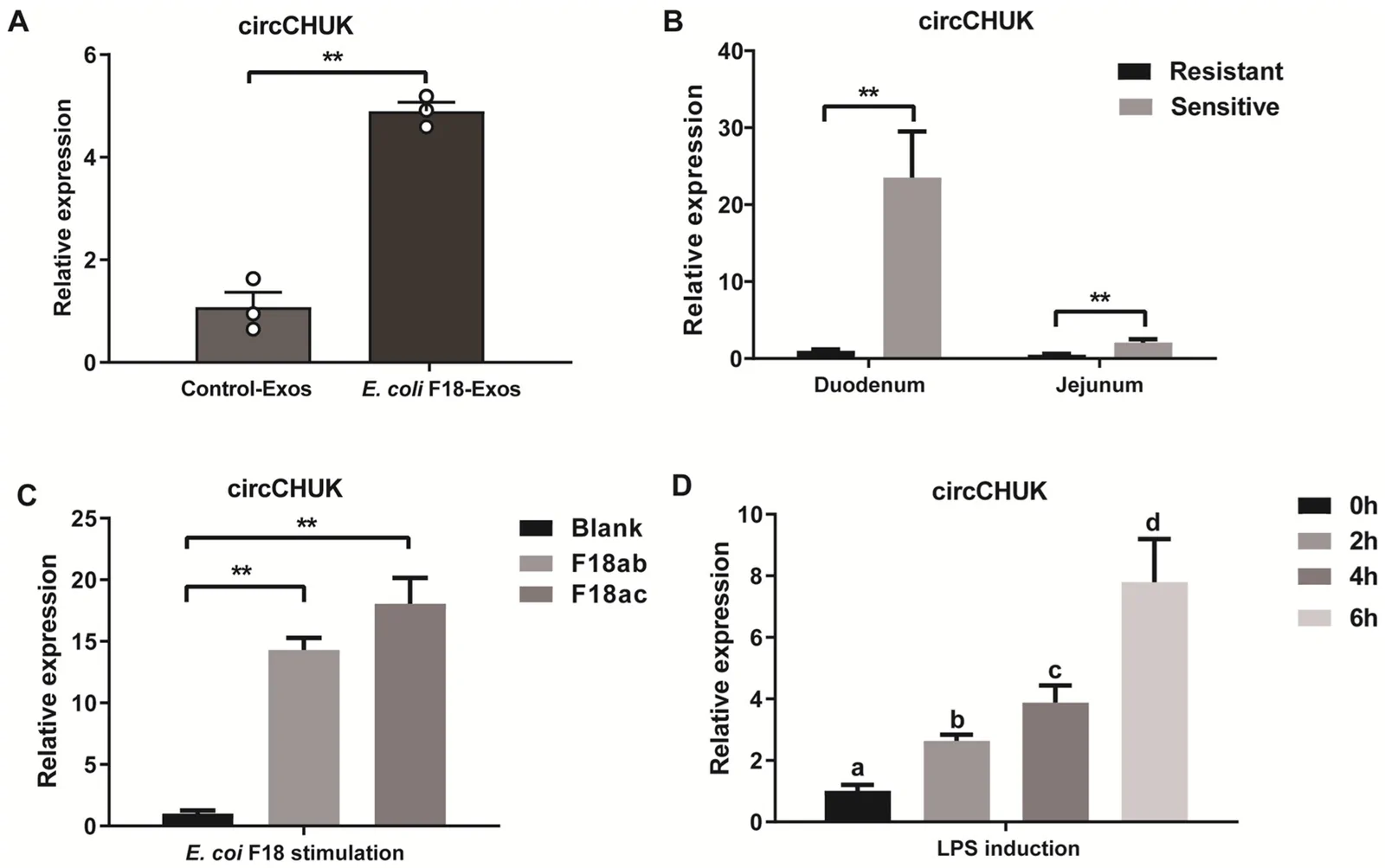
Linking circCHUK expression to *E. coli* F18 infection. (**A**) circCHUK levels in exosomes from *E. coli* F18-infected versus control IPEC-J2 cells. (**B**) circCHUK expression in duodenal and jejunal tissues from *E. coli* F18-sensitive and -resistant piglets. (**C**) Changes in circCHUK transcript levels in F18ab/ac-treated IPEC-J2 cells measured by qPCR. (**D**) Time-course changes in circCHUK expression in IPEC-J2 cells treated with 0.1 μg/mL LPS at 0, 2, 4, and 6 h. Distinct lowercase superscript letters denote statistically significant differences (*p* < 0.05). Values are expressed as mean ± SD; ** *p* < 0.01.

circRNA_07026 (circCHUK) was selected from the differentially expressed circRNAs identified between control and *E. coli* F18-infected IPEC-J2-derived exosomes because it exhibited the largest expression change (log_2_FoldChange = 11.03, *p* = 0.000316). We first confirmed that circCHUK expression was significantly increased in exosomes derived from *E. coli* F18-infected IPEC-J2 cells compared with control exosomes (Figure 5A, *p* < 0.01).

To determine whether this expression pattern was also associated with *E. coli* F18 susceptibility in vivo, circCHUK expression was further examined in jejunal and duodenal tissues from three *E. coli* F18-resistant and three *E. coli* F18-sensitive Meishan piglets previously characterized in our earlier study [16]. circCHUK expression was significantly lower in both the jejunum and duodenum of resistant piglets than in the corresponding tissues of sensitive piglets (Figure 5B, *p* < 0.01).

Consistent with these in vivo observations, stimulation of IPEC-J2 cells with either *E. coli* F18ab or F18ac significantly increased circCHUK expression compared with untreated cells (Figure 5C, *p* < 0.01). LPS treatment also induced a time-dependent increase in circCHUK expression at 2, 4, and 6 h (Figure 5D, *p* < 0.05). Together, these findings indicate that elevated circCHUK expression is associated with increased susceptibility to *E. coli* F18 and support the use of circCHUK as a candidate regulator for subsequent functional analysis.

**Figure 6:**
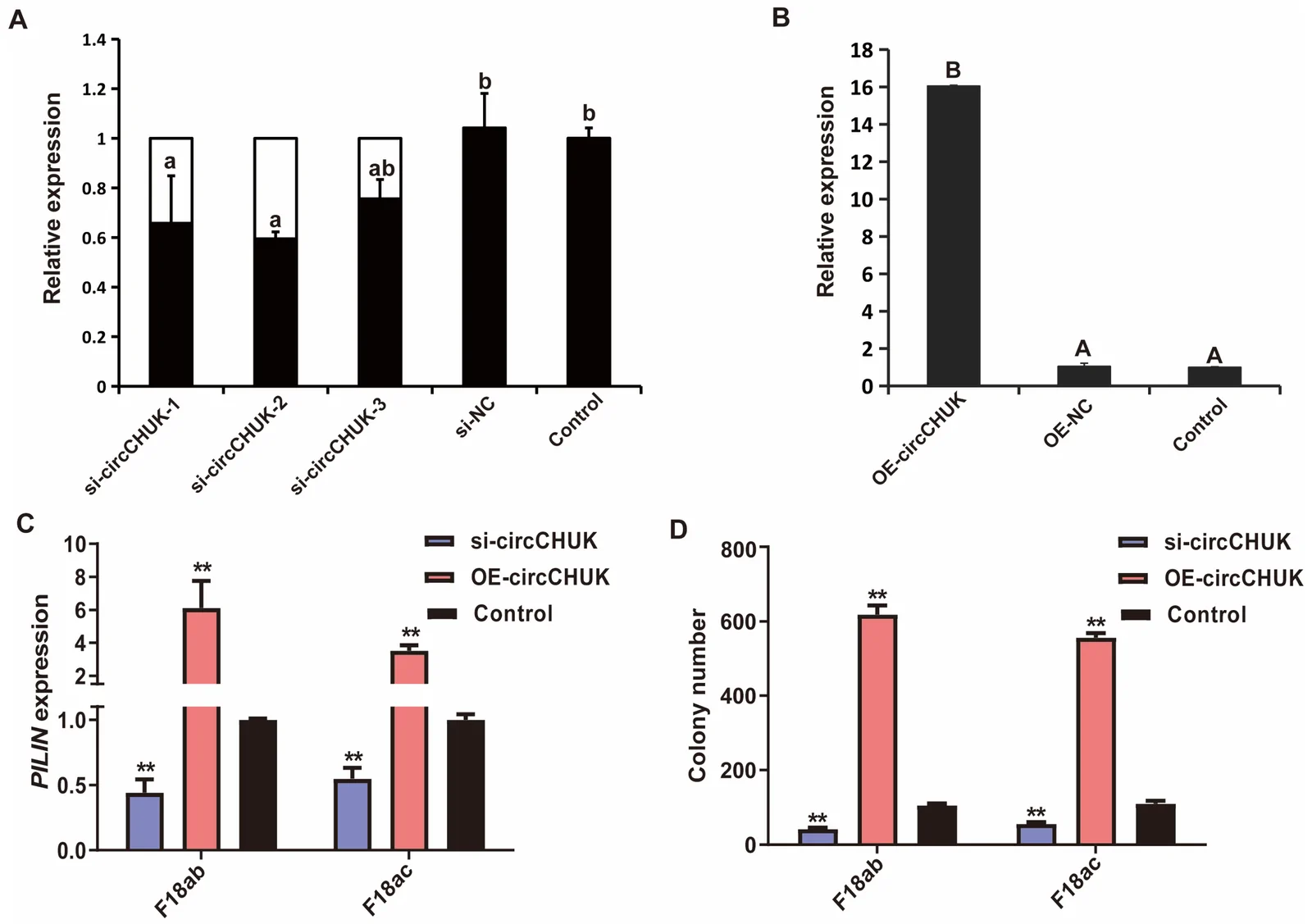
Influence of circCHUK on *E. coli* F18 adhesion to IPEC-J2 cells. (**A**) Efficiency of circCHUK knockdown in IPEC-J2 cells confirmed by qRT-PCR. (**B**) Efficiency of circCHUK overexpression in IPEC-J2 cells confirmed by qRT-PCR. (**C**) Expression of the *E. coli* F18 fimbrial gene *PILIN* in circCHUK-depleted and circCHUK-overexpressing IPEC-J2 cells. (**D**) Quantification of *E. coli* F18 fimbrial attachment to IPEC-J2 cells under each treatment condition. Distinct lowercase superscript letters indicate significant differences (*p* < 0.05). Distinct uppercase superscript letters indicate significant differences (*p* < 0.01). Data are shown as mean ± SD; ** *p* < 0.01.

To elucidate the potential correlation between circCHUK expression and *E. coli* F18 adhesion in IPEC-J2 cells, we evaluated whether alterations in circCHUK expression regulated F18-harboring fimbriae adhesion to IPEC-J2 cells. To do this, we designed an overexpression vector (OE-circCHUK) and 3 siRNA vectors (si-circCHUK-1, si-circCHUK-2, si-circCHUK-3) for circCHUK (Table S2). qPCR of si-circCHUK-transfected cells revealed a peak knockdown efficiency of 40% (Figure 6A). Alternatively, transfection with pcDNA3.1-circCHUK achieved a striking 16-fold rise in circCHUK transcript levels (Figure 6B). F18-fimbriae protein (PILIN) expression (Figure 6C) and colony number (Figure 6D) analyses further revealed a drastically declined proportion of F18 bacteria attached to the si-circCHUK IPEC-J2 cells (*p* < 0.01). Conversely, the bacterial population was significantly increased in OE-circCHUK IPEC-J2 cells (*p* < 0.01). Thus, both knockdown and overexpression of circCHUK markedly influenced the extent of *E. coli* F18 adhesion to IPEC-J2 cells, identifying circCHUK as a regulator of cellular susceptibility to this pathogen.

### 3.6. PPI Network Analysis of Target Genes and Screening of Key circRNA/miRNA/mRNA Axes

**Figure 7:**
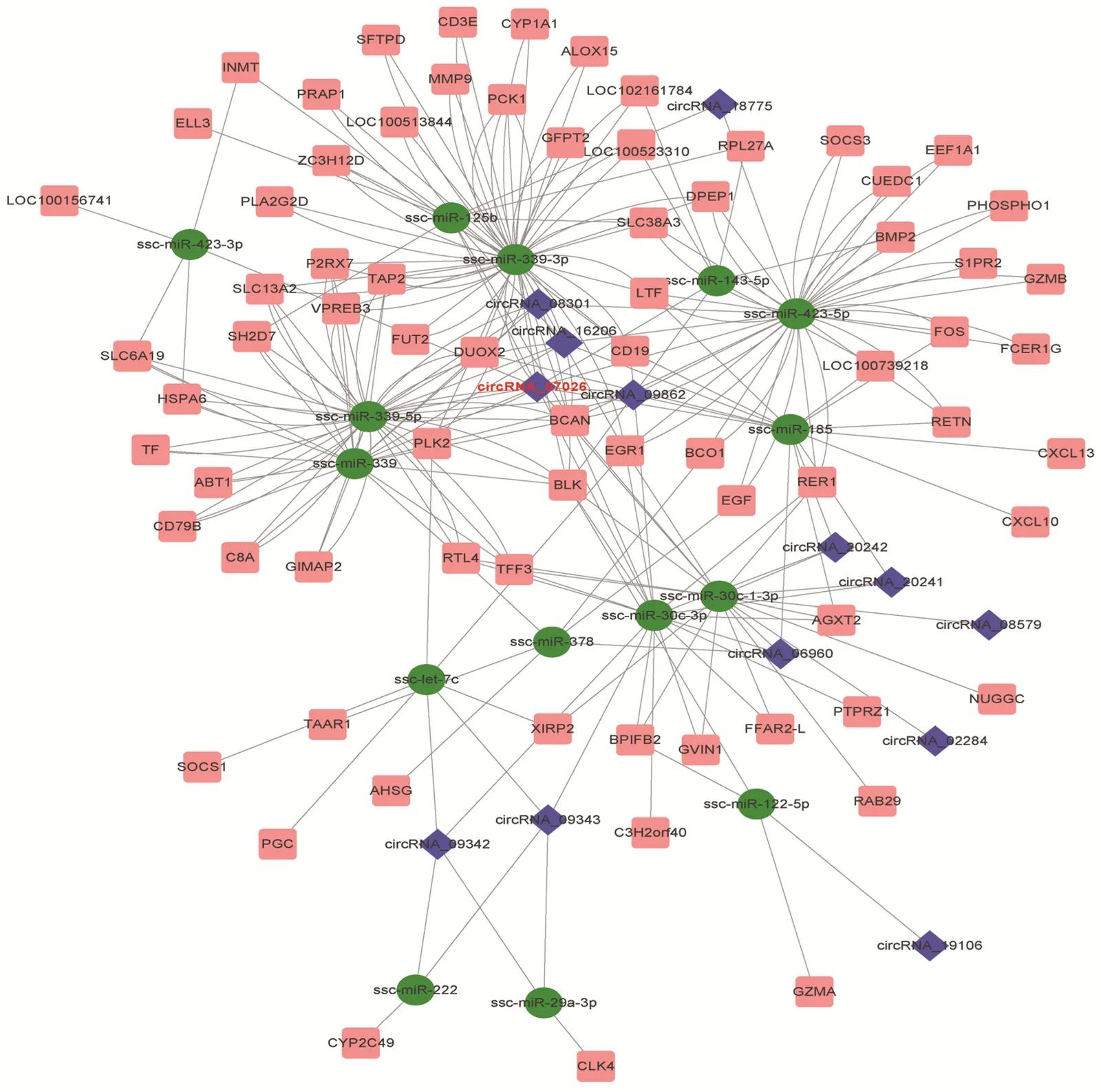
PPI network analysis of target genes and screening of circRNA/miRNA/mRNA axes. The network illustrates the predicted competing endogenous RNA (ceRNA) interactions of key circRNA (circRNA:13; miRNA:15; mRNA:73) visualized using Cytoscape. Red box represents the differential expression genes between *E. coli* F18-resistant and -sensitive piglets. Green box represents the differential expression miRNAs between *E. coli* F18-resistant and -sensitive piglets. Blue box represents the differential expression circRNAs in supernatant exosomes between normal IPEC-J2 and *E. coli* F18-infected IPEC-J2.

To investigate the downstream regulatory roles of circRNA, downstream targets of the DECs were explored by first predicting miRNA partners through miRDB and circNetVis, then mapping miRNA-mRNA interactions with miRWalk and TargetScan. These predictions were integrated with earlier differential miRNA and transcriptome datasets from *E. coli* F18-resistant versus -sensitive piglets [27] to build a ceRNA regulatory network in Cytoscape, comprising 13 circRNAs, 15 miRNAs, and 73 mRNAs (Figure 7, Table S6). circRNA_07026/circCHUK occupied the network’s core and connected 150 ceRNA nodes, among which the circCHUK/ssc-miR-339-3p/FUT2 triplet stood out. FUT2 has been proposed as a candidate target for reducing *E. coli* F18 susceptibility in weaned piglets [28]. Combining these reports with our network results points to the exo-circCHUK/ssc-miR-339-3p/FUT2 axis as a mechanism through which exosomal circCHUK may counter *E. coli* F18 susceptibility in IPEC-J2 cells.

## 4. Discussion

Understanding how exosomal circRNAs regulate IPEC-J2 cells after *E. coli* F18 exposure is a prerequisite for exploiting this cell line as an in vitro model of bacterial adhesion. Exosomes, the extracellular vesicles that cells release, are central to intercellular communication. By ferrying functional proteins, lipids, and RNA cargo to cells nearby or at distant sites, they carry out a range of biological tasks [29]. Yet what exosomes do when host cells are attacked by pathogens is still poorly understood. One area of growing activity concerns the role and mechanisms of host-cell-derived exosomes during PEDV infection [30]. IPEC-J2 cells are well suited for pathogenesis research because they are the direct target of *E. coli* F18. Their pronounced susceptibility to this pathogen has made them a standard system for studying bacterial adhesion and immune responses [27,31,32]. Accumulating data link exosomes to the cellular immune response against viruses and to the processes of viral infection, proliferation, and transmission [33,34,35]. In related work, Matsuno et al. mapped the mRNA content of exosome-like vesicles from porcine ovarian follicles [36], while a separate study showed that exosome-delivered miR-153 from *Trichinella spiralis* induces intestinal epithelial cell apoptosis through Bcl2 suppression [37]. These considerations make IPEC-J2 cells an appropriate model for investigating how *E. coli* F18 infection reshapes exosome properties and host transcriptome profiles.

Unlike their linear counterparts, circular RNAs (circRNAs) join their ends into a continuous, covalently closed loop. Once considered rare, these molecules are now recognized as a widespread and highly stable RNA class present across diverse eukaryotes, including humans, animals, and plants [7]. Exosomes routinely carry circRNAs among their cargo as they mediate crosstalk between cells, and multiple studies have documented the regulatory functions of exosomal circRNAs in lung adenocarcinoma [38], gastric cancer [39], and hepatocellular carcinoma [40]. These properties make exosomal circRNAs attractive candidates as disease indicators and prospective biomarkers. Wang et al. characterized the function of circLSM14A and demonstrated that non-coding RNAs delivered by milk exosomes participate in controlling PEDV replication [30]. The precise functions and mechanisms of exosomal circRNAs in IPEC-J2 cells during *E. coli* F18 infection, however, remain poorly understood and call for deeper investigation. In this study, RNA-seq was applied to map the circRNA content of exosomes from *E. coli* F18-infected and control cells. RNA-seq is well suited to genome-wide expression profiling, particularly for organisms whose genome sequences are incomplete. Previous studies have shown that porcine circRNAs are involved in several biological processes, including embryonic and skeletal muscle development [41,42], adipose deposition [43], skin development [11], metabolism-related traits [12], immune regulation [14], and responses to infectious diseases [30]. In this study, we detected 280 differentially expressed circRNAs (DECs) in IPEC-J2 exosomes associated with *E. coli* F18 susceptibility. KEGG analysis of parental genes for DECs pointed to enrichment in immune-related pathways. However, “Suckling behavior” appears among enriched biological-process terms for circRNA parental genes, which was related to a brief cautionary note about the possibility of enrichment noise in a modest parental-gene set. Among the upregulated and downregulated DECs, circCHUK exhibited the most pronounced change in expression. Little is known about the function of circCHUK and other pig circRNAs. Here, circCHUK was identified as one of the most strongly altered circRNAs in exosomes derived from *E. coli* F18-infected IPEC-J2 cells. Importantly, we further examined its expression in intestinal tissues from *E. coli* F18-resistant and -sensitive Meishan piglets to determine whether the in vitro expression pattern was also associated with susceptibility in vivo. circCHUK expression was significantly lower in both jejunal and duodenal tissues from resistant piglets than in tissues from sensitive piglets, whereas *E. coli* F18 stimulation markedly increased circCHUK expression in IPEC-J2 cells. These observations provide complementary in vivo and in vitro evidence linking circCHUK expression with *E. coli* F18 susceptibility. The Meishan pig intestinal tissues were used only for biological validation of the candidate circRNA identified from the cell-based exosomal transcriptome analysis, whereas the IPEC-J2 model was used for mechanistic experiments, including *E. coli* F18 stimulation, circCHUK knockdown and overexpression, and bacterial adherence assays. Taken together, these results identify exosomal circCHUK as a circRNA marker governing resistance to *E. coli* F18 infection.

Exosomes are intermediaries of intercellular crosstalk in metabolism. They reliably mirror changes in the cellular microenvironment and govern information transfer, microenvironmental remodeling, and immune regulation [44,45]. Mechanistic understanding of how exosomal circRNAs modulate *E. coli* F18-infected IPEC-J2 cells is needed before this system can be deployed as a reliable in vitro model of bacterial adhesion. While the molecular functions of circRNAs are still being mapped, certain exosomal circRNAs act as miRNA “sponges” with documented regulatory roles in human cancers [39] and pathogen infection [30]. Their involvement in ceRNA-based regulation during *E. coli* F18 infection, however, has yet to be established. Here, we demonstrated that α-(1,2)fucosyltransferase gene 2 (FUT2) is under strong control of the circCHUK/ssc-miR-339-3p axis. The pathogenicity of *E. coli* F18 depends on the expression of specific receptors at the brush border of piglet small intestinal epithelial cells [1]. The smallest antigenic determinant of the *E. coli* F18 receptor has been identified as the type 1 H antigen of the ABO blood group antigens [46], and the synthesis of this antigen is catalyzed by the enzyme encoded by FUT2 [47,48]. We therefore hypothesize that exosomal circCHUK acts as a central modulator of host susceptibility to *E. coli* F18 infection. The present study takes an initial step toward understanding exosomal circRNA function during *E. coli* F18 infection of IPEC-J2 cells, and we recommend that the proposed ceRNA mechanism be validated through in vivo experiments such as luciferase, RIP/pull-down, or expression assays. These results offer the first glimpse of how exosomal circRNAs regulate the cellular response to *E. coli* F18 infection, identify a candidate of practical value for application in weaned piglets, and provide conceptual guidance for molecular breeding aimed at diarrhea resistance in pigs.

## 5. Conclusions

Together, these results show that infection with *E. coli* F18 significantly alters both the mean particle size and the secretory output of exosomes. RNA-seq profiling of these vesicles uncovered 280 DECs, with circCHUK exhibiting the most striking difference. Exosomal circCHUK emerges as an important determinant of host-cell responses to *E. coli* F18: depleting or overexpressing it substantially changed the extent to which *E. coli* F18 adhered to IPEC-J2 cells. ceRNA network analysis pointed to the exo-circCHUK/ssc-miR-339-3p/FUT2 regulatory axis as a likely mediator of *E. coli* F18 infection. To our knowledge, this is the first account of the role and molecular basis of exosomal circRNAs in *E. coli* F18 infection, establishing a framework for dissecting the pathogenic mechanisms of *E. coli* F18 and for discovering new antibacterial targets.

## Data Availability

The raw data supporting the conclusions of this article will be made available by the corresponding author, without undue reservation.

## Supplementary Materials

The following supporting information can be downloaded at: https://www.mdpi.com/article/10.3390/ani16172684/s1, Table S1: Primer information for the RT-qPCR; Table S2: Sequence information of siRNA for circCHUK; Table S3: Statistics for the sequencing data; Table S4: Differentially expressed circRNAs between *E. coli* F18-exosomes and control-exosomes groups in IPEC-J2 (*E. coli* vs. NC); Table S5: List of KEGG pathways obtained by enrichment analysis; Table S6: ceRNA network analysis of circRNA.

## Abbreviations

PWD	post-weaning diarrhea
*E. coli* F18	*Escherichia coli* F18
circRNAs	Circular RNAs
miRNAs	microRNAs
IPEC-J2	intestinal porcine epithelial
ceRNA	competing endogenous RNA
Exos	exosomes
GO	Gene Ontology
KEGG	Kyoto Encyclopedia of Genes and Genomes
SD	standard deviation
IFA	indirect fluorescent antibody
DECs	differentially expressed circRNAs
LPS	lipopolysaccharide
FUT2	α-(1,2)fucosyltransferase gene 2

## References

[1] Boldin B. (2008). Persistence and spread of gastro-intestinal infections: The case of enterotoxigenic *Escherichia coli* in piglets. Bull. Math. Biol..

[2] Théry C., Witwer K.W., Aikawa E., Alcaraz M.J., Anderson J.D., Andriantsitohaina R., Antoniou A., Arab T., Archer F., Atkin-Smith G.K. (2018). Minimal information for studies of extracellular vesicles 2018 (MISEV2018): A position statement of the International Society for Extracellular Vesicles and update of the MISEV2014 guidelines. J. Extracell. Vesicles.

[3] Salvi V., Gianello V., Busatto S., Bergese P., Andreoli L., D’Oro U., Zingoni A., Tincani A., Sozzani S., Bosisio D. (2018). Exosome-delivered microRNAs promote IFN-alpha secretion by human plasmacytoid DCs via TLR7. JCI Insight.

[4] Tan L., Zhao M., Wu H., Zhang Y., Tong X., Gao L., Zhou L., Lu Q., Zeng J. (2021). Downregulated serum exosomal miR-451a expression correlates with renal damage and its intercellular communication role in systemic lupus erythematosus. Front. Immunol..

[5] Jeppesen D.K., Fenix A.M., Franklin J.L., Higginbotham J.N., Zhang Q., Zimmerman L.J., Liebler D.C., Ping J., Liu Q., Evans R. (2019). Reassessment of exosome composition. Cell.

[6] Cao Y., Wang Z., Yan Y., Ji L., He J., Xuan B., Shen C., Ma Y., Jiang S., Ma D. (2021). Enterotoxigenic *Bacteroides fragilis* promotes intestinal inflammation and malignancy by inhibiting exosome-packaged miR-149-3p. Gastroenterology.

[7] Zhou W.Y., Cai Z.R., Liu J., Wang D.S., Ju H.Q., Xu R.H. (2020). Circular RNA: Metabolism, functions and interactions with proteins. Mol. Cancer.

[8] Hong L., Gu T., He Y., Zhou C., Hu Q., Wang X., Zheng E., Huang S., Xu Z., Yang J. (2019). Genome-wide analysis of circular RNAs mediated ceRNA regulation in porcine embryonic muscle development. Front. Cell Dev. Biol..

[9] Yun J., Huang X., Liu C., Shi M., Li W., Niu J., Cai C., Yang Y., Gao P., Guo X. (2023). Genome-wide analysis of circular RNA-mediated ceRNA regulation in porcine skeletal muscle development. BMC Genom..

[10] Li B., Yang J., He J., Gong Y., Xiao Y., Zeng Q., Xu K., Duan Y., He J., Ma H. (2021). Spatiotemporal regulation and functional analysis of circular RNAs in skeletal muscle and subcutaneous fat during pig growth. Biology.

[11] Li Y., Shi R., Yuan R., Jiang Y. (2023). Comprehensive transcriptional analysis of pig facial skin development. PeerJ.

[12] Li A., Huang W., Zhang X., Xie L., Miao X. (2018). Identification and characterization of circRNAs of two pig breeds as a new biomarker in metabolism-related diseases. Cell. Physiol. Biochem..

[13] Chen J., Wang H., Jin L., Wang L., Huang X., Chen W., Yan M., Liu G. (2019). Profile analysis of circRNAs induced by porcine epidemic diarrhea virus infection in porcine intestinal epithelial cells. Virology.

[14] Wang Y., Cheng J., Xu C., Jin J., Bao W., Wu S., Wu Z. (2023). Genome-wide analysis of circRNA regulation during spleen development of Chinese indigenous breed Meishan pigs. BMC Genom..

[15] Peng O., Xia Y., Wei Y., Zeng S., Zou C., Hu F., Xu Q., Huang Y., Geng R., Hu G. (2023). Integrative transcriptomic profiling of mRNA, miRNA, circRNA, and lncRNA in alveolar macrophages isolated from PRRSV-infected porcine. Front. Immunol..

[16] Wu Z.C., Liu Y., Dong W.H., Zhu G.Q., Wu S.L., Bao W.B. (2016). CD14 in the TLRs signaling pathway is associated with the resistance to *E. coli* F18 in Chinese domestic weaned piglets. Sci. Rep..

[17] Wu H., Zhou J., Mei S., Wu D., Mu Z., Chen B., Xie Y., Ye Y., Liu J. (2017). Circulating exosomal microRNA-96 promotes cell proliferation, migration and drug resistance by targeting LMO7. J. Cell. Mol. Med..

[18] Yu Z., Xu X., Ai N., Wang K., Zhang P., Li X., LiuFu S., Liu X., Jiang J., Gu J. (2023). Integrated analysis of circRNA, lncRNA, miRNA and mRNA to reveal the ceRNA regulatory network of postnatal skeletal muscle development in Ningxiang pig. Front. Cell Dev. Biol..

[19] Nguyen T.H., Nguyen H.N., Vu T.N. (2024). CircNetVis: An interactive web application for visualizing interaction networks of circular RNAs. BMC Bioinform..

[20] Chen Y., Wang X. (2020). miRDB: An online database for prediction of functional microRNA targets. Nucleic Acids Res..

[21] Agarwal V., Bell G.W., Nam J.W., Bartel D.P. (2015). Predicting effective microRNA target sites in mammalian mRNAs. eLife.

[22] Shannon P., Markiel A., Ozier O., Baliga N.S., Wang J.T., Ramage D., Amin N., Schwikowski B., Ideker T. (2003). Cytoscape: A software environment for integrated models of biomolecular interaction networks. Genome Res..

[23] Xia P.P., Song Y.J., Zou Y.J., Yang Y., Zhu G.Q. (2015). F4+ enterotoxigenic *Escherichia coli* (ETEC) adhesion mediated by the major fimbrial subunit FaeG. J. Basic Microbiol..

[24] Dai C., Yang L., Jin J., Wang H., Wu S., Bao W. (2019). Regulation and Molecular Mechanism of TLR5 on Resistance to *Escherichia coli* F18 in Weaned Piglets. Animals.

[25] Dai C.H., Gan L.N., Qin W.U., Zi C., Zhu G.Q., Wu S.L., Bao W.B. (2016). Use of fluorescence quantitative polymerase chain reaction (PCR) for the detection of *Escherichia coli* adhesion to pig intestinal epithelial cells. Pol. J. Vet. Sci..

[26] Wang H., Gao X., Yu S., Wang W., Liu G., Jiang X., Sun D. (2022). Circular RNAs regulate parental gene expression: A new direction for molecular oncology research. Front. Oncol..

[27] Wu Z., Fan H., Jin J., Gao S., Huang R., Wu S., Bao W. (2022). Insight into mechanisms of pig lncRNA FUT3-AS1 regulating *E. coli* F18-bacterial diarrhea. PLoS Pathog..

[28] Wu Z., Feng H., Cao Y., Huang Y., Dai C., Wu S., Bao W. (2018). New insight into the molecular mechanism of FUT2 regulating *Escherichia coli* F18 resistance in weaned piglets. Int. J. Mol. Sci..

[29] Kalluri R., LeBleu V.S. (2020). The biology, function, and biomedical applications of exosomes. Science.

[30] Wang Y., Liu B., Liang J., Yang W., Wang H., Ouyang K., Luo J., Sun J., Xi Q., Zhang Y. (2025). CircLSM14A regulates PEDV replication via the miR-27b-5p/HMGB1 axis. Vet. Microbiol..

[31] Jin J., Liu M., Yu F., Sun M.A., Wu Z. (2024). METTL3 enhances *E. coli* F18 resistance by targeting IKBKG/NF-κB signaling via an m6A-YTHDF1-dependent manner in IPEC-J2 cells. Int. J. Biol. Macromol..

[32] Wu Z., Li M., Wu J., Jin S., Xu Y., Jin J., Wu Y. (2024). Characterization of the molecular role that ST3GAL1 plays in porcine susceptibility to *E. coli* F18 infection. Int. J. Biol. Macromol..

[33] Madison M.N., Okeoma C.M. (2015). Exosomes: Implications in HIV-1 pathogenesis. Viruses.

[34] Madison M.N., Jones P.H., Okeoma C.M. (2015). Exosomes in human semen restrict HIV-1 transmission by vaginal cells and block intravaginal replication of LP-BM5 murine AIDS virus complex. Virology.

[35] Saad M.H., Badierah R., Redwan E.M., El-Fakharany E.M. (2021). A comprehensive insight into the role of exosomes in viral infection: Dual faces bearing different functions. Pharmaceutics.

[36] Matsuno Y., Kanke T., Maruyama N., Fujii W., Naito K., Sugiura K. (2019). Characterization of mRNA profiles of the exosome-like vesicles in porcine follicular fluid. PLoS ONE.

[37] Wang R., Lin L., Han Y., Li Z., Zhen J., Zhang Y., Sun F., Lu Y. (2023). Exosome-delivered miR-153 from *Trichinella spiralis* promotes apoptosis of intestinal epithelial cells by downregulating Bcl2. Vet. Res..

[38] Zhang X., Xu Y., Ma L., Yu K., Niu Y., Xu X., Shi Y., Guo S., Xue X., Wang Y. (2022). Essential roles of exosome and circRNA_101093 on ferroptosis desensitization in lung adenocarcinoma. Cancer Commun..

[39] Deng C., Huo M., Chu H., Zhuang X., Deng G., Li W., Wei H., Zeng L., He Y., Liu H. (2024). Exosome circATP8A1 induces macrophage M2 polarization by regulating the miR-1-3p/STAT6 axis to promote gastric cancer progression. Mol. Cancer.

[40] Hu Z., Chen G., Zhao Y., Gao H., Li L., Yin Y., Jiang J., Wang L., Mang Y., Gao Y. (2023). Exosome-derived circCCAR1 promotes CD8+ T-cell dysfunction and anti-PD1 resistance in hepatocellular carcinoma. Mol. Cancer.

[41] Wang J., Chen J.F., Ma Q., Mo D.L., Sun J.J., Ren Q.L., Zhang J.Q., Lu Q.X., Xing B.S. (2022). Identification and characterization of circRNAs related to meat quality during embryonic development of the longissimus dorsi muscle in two pig breeds. Front. Genet..

[42] Li M., Zhang N., Li J., Ji M., Zhao T., An J., Cai C., Yang Y., Gao P., Cao G. (2023). CircRNA Profiling of Skeletal Muscle in Two Pig Breeds Reveals CircIGF1R Regulates Myoblast Differentiation via miR-16. Int. J. Mol. Sci..

[43] Chen Y., Hou X., Fu Y., Gao H., Wang Q., Fan N., Xie Q., Ren R., Chen F., Yin Y. (2025). Identification of candidate CircRNA associated with intramuscular fat deposition in Duroc pigs. Genomics.

[44] Anderson M.R., Kashanchi F., Jacobson S. (2016). Exosomes in viral disease. Neurotherapeutics.

[45] Isaac R., Reis F.C.G., Ying W., Olefsky J.M. (2021). Exosomes as mediators of intercellular crosstalk in metabolism. Cell Metab..

[46] Coddens A., Diswall M., Angström J., Breimer M.E., Goddeeris B., Cox E., Teneberg S. (2009). Recognition of blood group ABH type 1 determinants by the FedF adhesin of F18-fimbriated *Escherichia coli*. J. Biol. Chem..

[47] Meijerink E., Fries R., Vogeli P., Masabanda J., Wigger G., Stricker C., Neuenschwander S., Bertschinger H.U., Stranzinger G. (1997). Two α(1,2)-fucosyltransferase genes on porcine chromosome 6q11 are closely linked to the blood group inhibitor (S) and *Escherichia coli* F18 receptor (ECF18R) loci. Mamm. Genome.

[48] Vogeli P., Meijerink E., Fries R., Stricker C., Bertschinger H.U. (1997). A molecular test for the detection of *E. coli* F18 receptors: A breakthrough in the struggle against oedema disease and postweaning diarrhoea in swine. Schweiz. Arch. Tierheilkd..

